# Baicalein Attenuates Early Molecular and Proliferative Changes in MASH-Driven Hepatocarcinogenesis by Inhibiting the NLRP3 Inflammasome and Pyroptosis Pathway

**DOI:** 10.5812/ijpr-171126

**Published:** 2026-09-09

**Authors:** Jing Xu, Amirabas Bostani, Hongwei Qu

**Affiliations:** 1 Liver Transplant Center, Transplant Center, West China Hospital, Sichuan University Chengdu China; 2 Yerevan State Medical University After Mkhitar Heratsi Yerevan Armenia; 3 Department of Intervention, The Second Affiliated Hospital of Shaanxi University of Chinese Medicine Xianyang China

**Keywords:** NLRP3 Inflammasome, Pyroptosis, Flavonoids, Hepatocarcinogenesis, MASH

## Abstract

**Background:**

Metabolic dysfunction-associated steatohepatitis (MASH) is a major risk factor for hepatocellular carcinoma (HCC). The nucleotide-binding oligomerization domain-like receptor family pyrin domain-containing 3 (NLRP3) inflammasome and pyroptosis are key drivers of inflammation and tumorigenesis. Baicalein (BAI), a flavonoid derived from *Scutellaria baicalensis*, has anti-inflammatory and antitumor properties.

**Objectives:**

This study aimed to evaluate the effects of BAI on early molecular and proliferative changes in a MASH-driven hepatocarcinogenesis model, using validated surrogate markers of preneoplastic progression (Ki-67 and glutathione S-transferase pi 1 [GSTP1]) rather than direct histopathological tumor quantification.

**Methods:**

An M-HCC rat model was established using a hepatopathogenic diet, carbon tetrachloride, and diethylnitrosamine. Rats received BAI (50 mg/kg/day) orally for 16 weeks. Biochemical, oxidative stress, and fibrosis parameters were measured, and the expression of NLRP3 inflammasome components, inflammatory cytokines, and proliferation markers was assessed. The findings were validated in lipotoxic HepG2 cells treated with BAI, with or without the NLRP3 inhibitor MCC950.

**Results:**

BAI significantly reduced serum liver enzyme levels, oxidative stress, and fibrosis markers in M-HCC rats. BAI treatment was associated with reduced NLRP3 inflammasome activation and pyroptosis marker levels, as evidenced by decreased levels of NLRP3, apoptosis-associated speck-like protein containing a CARD (ASC), caspase-1, the N-terminal fragment of gasdermin D (GSDMD-N), IL-1β, and IL-18, together with reduced steatosis and proliferation markers. In lipotoxic HepG2 cells, BAI dose-dependently suppressed NLRP3 activation and pyroptosis; cotreatment with MCC950 did not further enhance these effects.

**Conclusions:**

BAI treatment was associated with suppression of components of the NLRP3 inflammasome and pyroptosis pathways, as well as reductions in inflammatory, fibrotic, and proliferative markers. These findings support further investigation of BAI as a candidate for modulating early hepatocarcinogenic events. However, direct anti-HCC efficacy remains to be established in future studies incorporating histological tumor endpoints.

## 1. Background

Metabolic dysfunction-associated steatohepatitis (MASH) is a severe form of metabolic liver disease characterized by lipid accumulation, lobular inflammation, and hepatocyte hypertrophy (1). MASH is an increasing global health concern and a major risk factor for hepatocellular carcinoma (HCC) (2). The pathological progression from uncomplicated steatosis to MASH and subsequently to HCC is driven by a complex, self-perpetuating cycle involving ongoing hepatic injury, inflammatory responses, and dysregulated tissue regeneration (3). Within this pathological milieu, the nucleotide-binding oligomerization domain-like receptor family pyrin domain-containing 3 (NLRP3) inflammasome has been recognized as a critical signaling hub that amplifies inflammatory pathways and promotes disease progression (4).

Activation of the NLRP3 inflammasome in hepatocytes and hepatic immune cells, often triggered by the lipotoxicity and oxidative stress inherent to MASH, promotes the autocatalytic conversion of procaspase-1 to its active form (5). Activated caspase-1 drives two major proinflammatory processes: the proteolytic maturation and secretion of key pro-inflammatory cytokines, such as interleukin-1β (IL-1β) and IL-18, and the cleavage of gasdermin D (GSDMD), which instigates a lytic form of programmed cell death referred to as pyroptosis (6, 7). Moreover, oxidative stress is recognized as a major contributor to the induction of cell death in hepatic cells (8).

Currently, few pharmacological treatments effectively prevent or reverse progression from MASH to HCC. This situation underscores the critical need to identify new therapeutic agents capable of modulating this key inflammatory cascade (9, 10). Natural flavonoids, recognized for diverse biological actions across multiple targets and broad pleiotropic effects, represent a promising reservoir of potential therapeutic agents (11). Baicalein (BAI), 5,6,7-trihydroxyflavone, is a notable flavonoid compound that has exhibited anti-inflammatory, antioxidant, and anti-cancer properties in diverse preclinical studies (12, 13). Prior research has demonstrated BAI's capacity to mitigate hepatic fibrosis and impede the proliferation of existing HCC cells. In addition, recent findings indicate that BAI can suppress NLRP3 inflammasome activation in contexts of metabolic dysfunction-related fatty liver disease (MASLD) and other inflammatory states (14, 15).

Nevertheless, the efficacy of BAI in MASH-driven hepatocarcinogenesis, and the precise molecular mechanisms by which it interacts with the NLRP3 inflammasome and the pyroptosis pathway, warrant further investigation. It remains unclear whether BAI can disrupt the critical interplay between inflammation and cell death that drives the transition from steatohepatitis to malignancy.

## 2. Objectives

This study tested the hypothesis that BAI attenuates early molecular and proliferative changes associated with MASH-driven hepatocarcinogenesis, as assessed using the surrogate markers Ki-67 and GSTP1, by inhibiting the NLRP3 inflammasome and the associated pyroptosis pathway.

## 3. Methods

### 3.1. Animals and Housing

Six-week-old male Fischer 344 rats (weight 100 - 120 g) were obtained from the Experimental Animal Center of Shaanxi University of Chinese Medicine. Upon arrival, the rats were acclimatized for one week under controlled conditions (temperature, 21 ± 1 °C; relative humidity, 50 - 60%; 12:12-hour light/dark cycle, lights on at 07:00). Animals were housed in groups of two in individually ventilated cages with standard environmental enrichment (nesting material and red polycarbonate tunnels). Standard rodent chow (5053, LabDiet) and tap water were provided ad libitum. Daily welfare monitoring included body weight measurement, assessment of general behavior, and evaluation for signs of pain or distress (e.g., piloerection, hunched posture, lethargy). Predefined humane endpoints were body weight loss exceeding 20% within one week, inability to reach food or water, persistent lethargy unresponsive to stimulation, or abdominal distension suggestive of ascites. No animals reached any humane endpoint, and no spontaneous deaths or adverse events attributable to BAI administration occurred during the 16-week study protocol.

### 3.2. Experimental Design

The experimental unit for all in vivo analyses was the individual rat. Six-week-old male Fischer 344 rats (n = 32, weight 100 - 120 g) were randomly assigned to four groups (n = 8 per group): 1) CTL (standard diet + vehicle injections), 2) BAI (standard diet + baicalein 50 mg/kg/day by gavage), 3) M-HCC (hepatopathogenic diet + CCl_4_ and DEN injections), and 4) M-HCC+BAI (same as M-HCC plus baicalein 50 mg/kg/day). The study duration was 16 weeks. The hepatopathogenic diet was provided from week 0 to week 16. Carbon tetrachloride (CCl_4_; 400 mg/kg body weight; catalog No. 289116, Merck-Millipore, Burlington, MA, USA) was diluted in liquid petrolatum (7110; Reactivos Química Meyer, Mexico City, Mexico) and administered intraperitoneally (i.p.) at a volume of 2 mL/kg body weight once weekly from week 0 to week 14 (15 injections). Diethylnitrosamine (DEN; 40 mg/kg body weight; catalog No. N0756, Merck-Millipore) was dissolved in Milli-Q water and administered i.p. at a volume of 2 mL/kg body weight once weekly from week 7 to week 16 (10 injections). Baicalein (BAI; catalog No. B9753, Merck-Millipore) was suspended in 0.7% carboxymethyl cellulose (5895 - 1000, Reactivos Química Meyer) and administered daily by oral gavage at a dose of 50 mg/kg body weight in a volume of 5 mL/kg from week 0 to week 16. Control rats received equivalent volumes of the respective vehicles (liquid petrolatum for CCl_4_, Milli-Q water for DEN, and 0.7% carboxymethyl cellulose for BAI) (Table 1). At week 17 (one week after the last BAI administration), all rats were euthanized. The following outcomes were assessed: serum liver enzymes (ALT, AST, GGT, ALP) and hyaluronic acid (at euthanasia); hepatic oxidative stress markers (MDA, SOD, 8-OHdG); and protein/mRNA expression of NLRP3 pathway components, inflammatory cytokines, fibrosis markers, steatosis markers, and proliferation markers (all from liver tissue harvested at necropsy). A timeline schematic is provided in Figure 1.

**Table 1.** Hepatopathogenic Diet

Ingredients	Percentage	Catalog	Brand
**Casein**	10.0	–	Irish Dairy Board, Dublin, IE
**Cholate**	0.5	C1254	Merck-Millipore, Burlington, MA, USA
**Cholesterol**	1.0	C8667	Merck-Millipore, Burlington, MA, USA
**Icing sugar**	30.0	–	Zulka (Kuoamex S.A. de C.V., Culiacán, SIN, MX)
**Unsalted butter**	5.0	–	Gloria (Cremería Americana S.A. de C.V., Mexico City, CMX, MX)
**Standard 5053 diet**	53.5	5053	LabDiet, Brentwood, MO, USA

**Figure 1.**
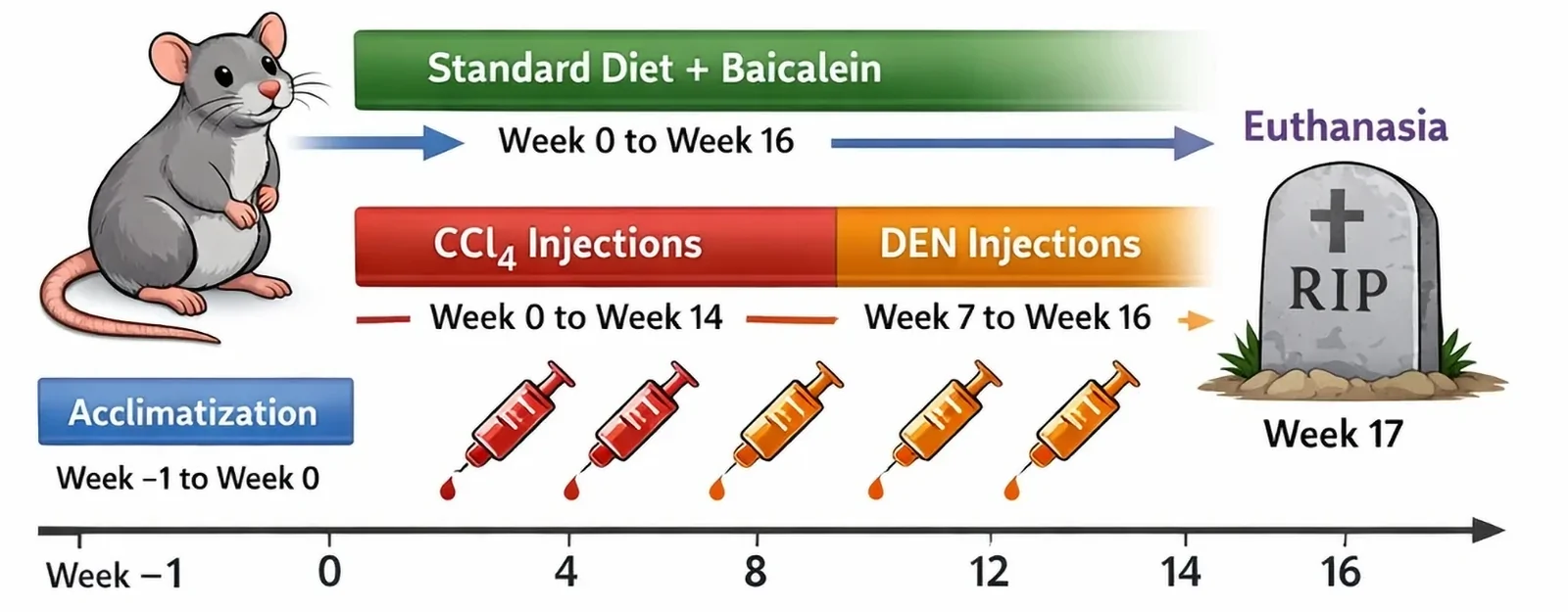
Schematic timeline of the experimental protocol. Following one week of acclimatization (Week −1 to 0), rats were randomly allocated to four experimental groups (CTL, BAI, M-HCC, and M-HCC+BAI; n = 8 per group). From Week 0 to Week 16, animals received either a standard diet (CTL and BAI) or a hepatopathogenic diet (M-HCC and M-HCC+BAI). Baicalein (50 mg/kg/day, oral gavage) was administered throughout the 16‑week experimental period. In the M-HCC and M-HCC+BAI groups, hepatocarcinogenesis was induced by weekly CCl_4_ injections (400 mg/kg) from Week 0 to Week 14, while DEN injections (40 mg/kg) were initiated at Week 7 and continued for 10 weeks. Animals were euthanized at Week 17 for tissue collection and downstream analyses.

The sample size (n = 8 per group) was based on previously published studies using similar MASH-driven hepatocarcinogenesis models (16, 17). No animals were excluded or died during the 16‑week study. The prespecified primary outcome was inhibition of NLRP3 inflammasome activation and pyroptosis, assessed by hepatic protein levels of NLRP3, ASC, cleaved caspase-1, GSDMD-N, IL-1β, and IL-18. Secondary outcomes included serum liver enzymes (ALT, AST, GGT, ALP) as markers of hepatic injury; oxidative stress markers (MDA, SOD, 8-OHdG); fibrosis markers (HA, TGF-β, α-SMA, collagen I); steatosis markers (SREBP-1c, PPARγ); and proliferation markers (Ki-67, GSTP1). All outcomes were measured at the end of the study. This study did not perform direct histopathological quantification of HCC nodules or tumors. Instead, hepatocarcinogenesis progression was assessed using molecular surrogate markers: the proliferation marker Ki-67 and the preneoplastic marker GSTP1. The M-HCC model used here has been previously characterized to produce HCC at 16 - 18 weeks (17); however, our endpoints were limited to preneoplastic and proliferative changes.

### 3.3. Randomization and Blinding

Rats were randomly assigned to the four experimental groups using a computer-generated random number sequence (simple randomization, no stratification or blocking). Allocation concealment was maintained using sequentially numbered, opaque, sealed envelopes, which were opened only immediately before the first intervention. The investigator responsible for daily gavage administration of BAI or vehicle was blinded to group allocation. All serum biochemical measurements (ALT, AST, GGT, ALP), ELISA assays (for NLRP3, ASC, cleaved caspase-1, GSDMD, GSDMD-N, IL-1β, IL-18, TGF-β, α-SMA, collagen I, SREBP-1c, PPARγ, Ki-67, GSTP1), and qPCR analyses were performed by an investigator blinded to the treatment groups. Data analysis was also conducted in a blinded manner until all results were finalized.

### 3.4. Biochemical Measurements

Blood samples were collected by intracardiac puncture at the time of euthanasia and centrifuged at 3000 rpm for 15 min to separate serum. To assess liver injury, serum activities of alanine aminotransferase (ALT), aspartate aminotransferase (AST), alkaline phosphatase (ALP), and γ-glutamyl transpeptidase (GGT) were measured using standardized colorimetric methods. To evaluate oxidative stress, a key driver of NLRP3 inflammasome activation, hepatic malondialdehyde (MDA) content, a marker of lipid peroxidation, was measured in liver homogenates using the thiobarbituric acid reactive substances (TBARS) assay. In addition, the activity of the antioxidant enzyme superoxide dismutase (SOD) was determined in liver tissue using a commercially available kit (19160, Sigma-Aldrich, St. Louis, MO, USA) according to the manufacturer’s protocol. Absorbance measurements were performed at 515 nm for ALT, 505 nm for AST, 410 nm for ALP and GGT, and 532 nm for MDA. Milli-Q water was used as a reference blank, and all spectrophotometric readings were obtained using a SHIMADZU UV-1201 spectrophotometer (Shimadzu Corporation, Kyoto, JPN). To complement the assessment of fibrosis, serum levels of the fibrogenesis marker hyaluronic acid (HA) were quantified using a specific ELISA kit (DHYAL0, R&D Systems, Minneapolis, MN, USA). To evaluate oxidative DNA damage, the marker 8‑hydroxy‑2′‑deoxyguanosine (8-OHdG) was quantified in liver tissue homogenates using a commercially available competitive ELISA kit (ab201734, Abcam, Cambridge, UK) according to the manufacturer’s instructions. Absorbance was measured at 450 nm, and 8-OHdG concentration was expressed as pg per mg of total protein.

### 3.5. Gene Expression Analysis by Quantitative Real-Time PCR (qPCR)

Total RNA was extracted from approximately 30 mg of liver tissue using TRIzol reagent (15596026, Invitrogen, Carlsbad, CA, USA) according to the manufacturer’s protocol. RNA concentration and purity (260/280 nm ratio ~2.0) were determined using a NanoDrop spectrophotometer (Thermo Fisher Scientific, Waltham, MA, USA). First-strand cDNA was synthesized from 1 µg of total RNA using a High-Capacity cDNA Reverse Transcription Kit (4368814, Applied Biosystems, Foster City, CA, USA). Quantitative real-time PCR was performed using PowerUp SYBR Green Master Mix (A25742, Applied Biosystems) on a QuantStudio 3 Real-Time PCR System (Applied Biosystems). The mRNA expression of the following genes was analyzed: Nlrp3, Pycard (ASC), Caspase-1, Gsdmd. Gene-specific primer pairs (Table 2) were designed using Primer-BLAST (NCBI) and synthesized by Integrated DNA Technologies (Coralville, IA, USA). Relative expression of each target gene was normalized to the housekeeping gene Gapdh and calculated using the 2^–ΔΔCT^ method.

**Table 2.** Primer Sequences for Quantitative Real-Time PCR

Genes	Forward Primer (5' → 3')	Reverse Primer (5' → 3')
**Nlrp3**	AGAGCACCTGTTGTGCAAGA	CAGGTTGCTGGTCTTGAAGG
**Pycard**	GCTGCCAAGACCTTCACCTA	GTCTGCCTGGTAGGTGTTCC
**Caspase-1**	ACAAGACCTGTGTCCTGTCC	TCCCACAAAGTCATCCATCC
**Gsdmd**	GCTGCTGGATGAAGACTGTG	CTGCAGCTTGATGTCGTAGG
**Gapdh**	AGGTCGGTGTGAACGGATTTG	TGTAGACCATGTAGTTGAGGTCA

### 3.6. Protein Expression Analysis by Enzyme-Linked Immunosorbent Assay (ELISA)

Protein levels of targets related to inflammasome activation, pyroptosis, fibrosis, steatosis, and proliferation were quantified in liver tissue homogenates using enzyme-linked immunosorbent assay (ELISA). All liver tissue samples were obtained from the left lateral lobe to ensure anatomical consistency. Samples were homogenized in ice-cold RIPA buffer supplemented with protease and phosphatase inhibitors (P8340 and P0044, Merck-Millipore, Burlington, MA, USA). Homogenization was performed using a PowerLyzer 24 tissue homogenizer (Qiagen, Hilden, Germany) at 4°C for 3 cycles of 30 seconds at 5,000 rpm, with 30-second pauses on ice between cycles. The total protein concentration of the homogenates was determined using the bicinchoninic acid (BCA) method. All ELISA assays were performed in duplicate. The intra-assay coefficient of variation (CV) was < 8%, and the inter-assay CV was < 10% for all kits. Standard curves were generated for each plate with R^2^ values > 0.99. Commercially available rat-specific ELISA kits were used according to the manufacturers' protocols to measure hepatic levels of the following proteins: NLRP3 (LS-F40205, LifeSpan BioSciences, Seattle, WA, USA), apoptosis-associated speck-like protein containing a CARD (ASC; MBS2603201, MyBioSource, San Diego, CA, USA), active caspase-1 (MBS2602305, MyBioSource), gasdermin D (GSDMD; MBS2882748, MyBioSource), the N-terminal fragment of GSDMD (GSDMD-N; MBS9697529, MyBioSource), interleukin-1 beta (IL-1β; ab255730, Abcam, Cambridge, UK), interleukin-18 (IL-18; ab216165, Abcam), transforming growth factor-beta (TGF-β; DB100B, R&D Systems, Minneapolis, MN, USA), alpha-smooth muscle actin (α-SMA; ab277297, Abcam), collagen type I (MBS2603354, MyBioSource), Ki-67 (MBS2702298, MyBioSource), glutathione S-transferase pi 1 (GSTP1; MBS764642, MyBioSource), sterol regulatory element-binding protein 1c (SREBP-1c; MBS2603202, MyBioSource), and peroxisome proliferator-activated receptor gamma (PPARγ; MBS2604166, MyBioSource). Absorbance was measured at 450 nm using a microplate reader (ELx808, BioTek Instruments, Winooski, VT, USA). Protein concentrations in liver homogenates were normalized to the total protein content and expressed as µg or ng per mg of total protein. The investigator performing ELISA measurements was blinded to treatment group allocation.

### 3.7. In Vitro Cell Culture and Treatments

Human hepatocellular carcinoma HepG2 cells (ATCC® HB-8065™) were cultured in Dulbecco's Modified Eagle Medium (DMEM; D6429, Merck-Millipore, Burlington, MA, USA) supplemented with 10% fetal bovine serum (FBS; 10437028, Thermo Fisher Scientific, Waltham, MA, USA) and 1% penicillin-streptomycin (P4333, Merck-Millipore) at 37°C in a humidified atmosphere containing 5% CO_2_. The experimental unit was an independent biological replicate (each representing a separate passage of HepG2 cells on a different day). Six independent biological replicates were performed (n = 6), and each replicate was run in technical triplicate for all assays. To mimic lipotoxic stress characteristic of MASH, cells were exposed to a lipotoxic cocktail consisting of 0.5 mM sodium palmitate (P9767, Merck-Millipore) and 1.0 mM sodium oleate (O7501, Merck-Millipore) for 24 hours. Cells were treated with BAI (B9753, Merck-Millipore) at concentrations of 20, 40, and 80 µM, either alone or in combination with the selective NLRP3 inflammasome inhibitor MCC950 (10 µM; 5381200001, Merck-Millipore). BAI concentrations (20, 40, 80 µM) were selected based on preliminary MTT cytotoxicity assays, which demonstrated no significant reduction in cell viability up to 100 µM, and are consistent with previously reported concentrations in hepatic cell studies (14, 15). MCC950 was used at 10 µM, a concentration widely reported to selectively inhibit NLRP3 inflammasome activation in vitro. Cell viability was assessed using the MTT assay. Protein was harvested for subsequent analysis of NLRP3 pathway components by ELISA. Quantification of MTT absorbance and ELISA results was performed by an investigator blinded to the treatment conditions, as detailed in the Randomization and Blinding subsection.

### 3.8. Statistical Analysis

All data are expressed as mean ± SD. For the in vivo and in vitro studies, statistical comparisons among experimental groups were performed using one-way analysis of variance (ANOVA) followed by Tukey's post hoc test for multiple comparisons. Normality was assessed using the Shapiro–Wilk test and homoscedasticity using Levene's test; no violations were detected. All in vivo assays included 8 biological replicates (n = 8 per group). qPCR was performed in technical triplicates for each biological replicate, and no animals were excluded. For significant ANOVA results, effect estimates for the primary between‑group comparisons are reported together with their corresponding 95% confidence intervals (CIs) in the Results section and/or relevant Supplementary Tables. The threshold for statistical significance was set at P < 0.05. The primary outcome (NLRP3 inflammasome inhibition) was prespecified and tested without adjustment for multiple comparisons. All secondary outcomes (liver enzymes, oxidative stress, fibrosis, steatosis, and proliferation markers) were analyzed as exploratory endpoints; therefore, no correction for multiple testing (e.g., Bonferroni) was applied, and p values are interpreted descriptively. All analyses were conducted using GraphPad Prism software, version 8.0.1 (GraphPad Software, La Jolla, CA, USA).

## 4. Results

### 4.1. BAI Protects Against Hepatic Injury and Oxidative DNA Damage in the M-HCC Model

Induction of the M-HCC model resulted in substantial hepatic injury, as evidenced by a marked elevation in serum ALT activity compared with the CTL group (approximately 4.6-fold; 95% CI 137.6 - 162.7; P < 0.001; Figure 2A). This hepatic injury was significantly mitigated by BAI treatment, with the M-HCC+BAI group exhibiting a 45% decrease in ALT concentrations relative to the M-HCC group (95% CI −100.3 to −72.5; P < 0.001). A comparable trend was observed for AST, with M-HCC induction resulting in an almost 3.5-fold increase (95% CI 185.4 - 250.9; P < 0.001 vs. CTL; Figure 2B); this elevation was reduced by 43% following BAI treatment (95% CI −167.1 to −96.6; P < 0.001 vs. M-HCC). In addition, serum activities of the cholestatic biomarkers GGT and ALP were significantly increased in the M-HCC group (approximately 5.9-fold and 1.7-fold, respectively; GGT 95% CI 16.3 - 23.7; ALP 95% CI 79.82 - 114.3; both P < 0.001 vs. CTL; Figure 2C, D). BAI administration significantly reduced these elevations by 65.7% for GGT and 31.8% for ALP (GGT 95% CI −19.49 to −12.09; ALP 95% CI −90.38 to −55.87; both P < 0.001 vs. M-HCC). Although minor numerical increases were observed in AST, GGT, and ALP in the BAI-alone group compared with CTL, these changes were small in magnitude and did not indicate overt hepatic injury (Table S1 in the Supplementary File).

**Figure 2.**
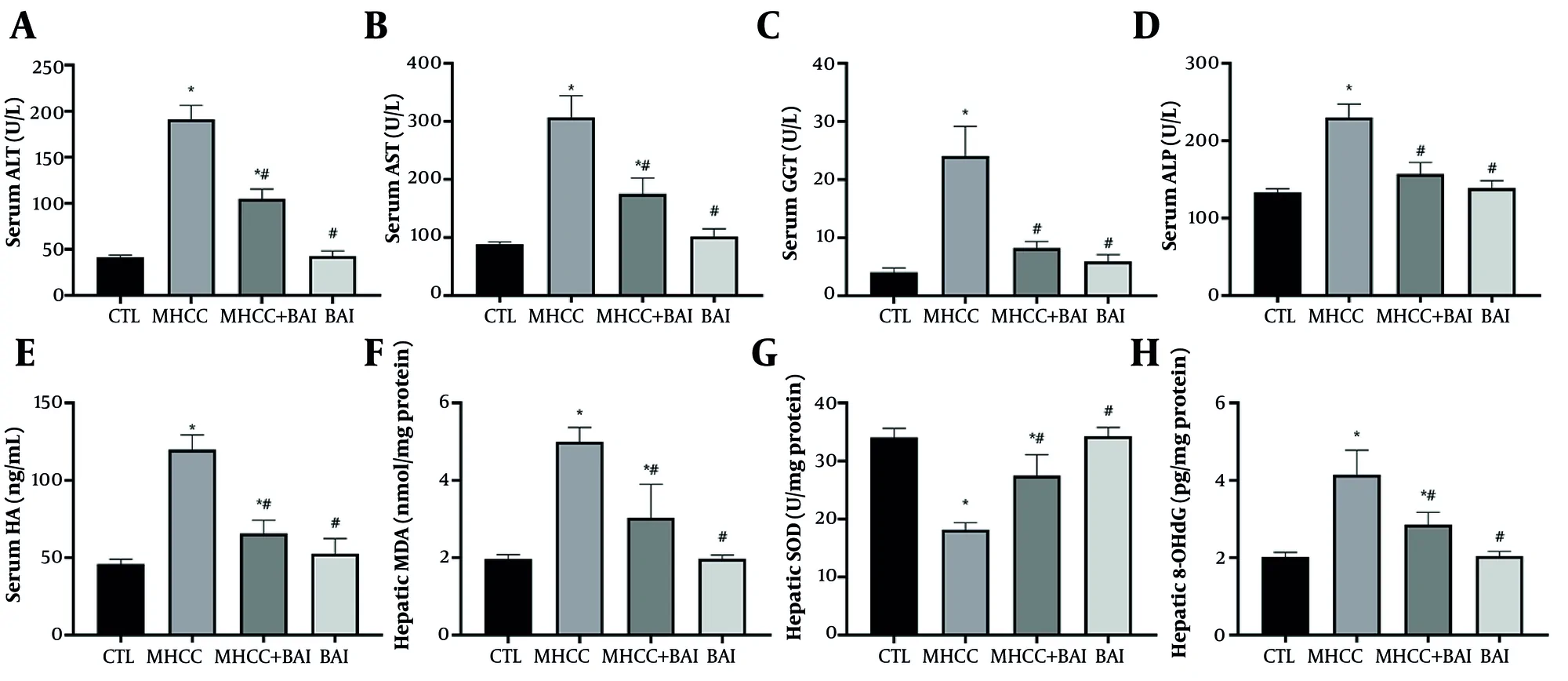
BAI attenuates hepatic injury, fibrosis, and oxidative stress in the MASH-driven HCC model. (A–D) Serum activities of alanine aminotransferase (ALT), aspartate aminotransferase (AST), γ-glutamyl transpeptidase (GGT), and alkaline phosphatase (ALP). (E) Serum concentration of the fibrogenesis marker hyaluronic acid (HA). (F) Hepatic malondialdehyde (MDA) content, a marker of lipid peroxidation. (G) Hepatic superoxide dismutase (SOD) activity. (H) Hepatic levels of 8-hydroxy-2′-deoxyguanosine (8-OHdG), a marker of oxidative DNA damage. Data are presented as mean ± SD (n = 8 per group). Statistical significance was determined by one-way ANOVA followed by Tukey's post hoc test. *: Significant difference vs. CTL; #: Significant difference vs. M-HCC; *#: Significant difference vs. both CTL and M-HCC groups. P < 0.05 was considered significant.

Consistent with the development of a fibrotic microenvironment, serum levels of HA were 2.61 times higher in the M-HCC group than in CTL rats (95% CI 62.71 - 85.12; P < 0.001; Figure 2E). BAI significantly decreased serum HA levels by 45.2% compared with the M-HCC group (95% CI −65.34 to −42.93; P < 0.001). Oxidative stress was markedly increased in the livers of M-HCC rats. Hepatic MDA content was 2.55 times higher in the M-HCC group than in CTL rats (95% CI 2.38 - 3.68; P < 0.001; Figure 2F). In addition, the activity of the antioxidant enzyme SOD was significantly decreased in the livers of M-HCC rats compared with CTL rats (95% CI −18.9 to −12.9; P < 0.001; Figure 2G). BAI treatment partially normalized oxidative stress markers, significantly reducing hepatic MDA compared with M-HCC (95% CI −2.61 to −1.31; P < 0.001) and increasing SOD activity (95% CI 6.3 - 12.3; P < 0.001) toward CTL levels, whereas BAI alone did not differ from CTL for either marker. Consistent with these changes in oxidative stress, hepatic 8-OHdG levels were markedly higher in M-HCC than in CTL rats (95% CI 1.63 - 2.63; P < 0.001; Figure 2H) and were significantly reduced by BAI in the M-HCC+BAI group (95% CI −1.78 to −0.79; P = 0.0001), whereas 8-OHdG levels in the BAI group were comparable to CTL (95% CI −0.53 to 0.47; P = 0.9986; Table S2 in the Supplementary File).

### 4.2. BAI Inhibits NLRP3 Inflammasome Activation and Pyroptosis in Vivo

Induction of the M-HCC model was associated with significant activation of the NLRP3 inflammasome, as indicated by substantially higher protein levels of NLRP3 (P < 0.001; Figure 3A), ASC (P < 0.001; Figure 3B), and cleaved caspase-1 (P < 0.001; Figure 3C). Moreover, activation of the effector molecule of pyroptosis, GSDMD, was observed, with a 3.3-fold higher protein level than in CTL (P < 0.001; Figure 3D). The active form of GSDMD, its N-terminal fragment (GSDMD-N) that forms membrane pores, was also significantly higher (P < 0.001; Figure 3E). Daily administration of BAI significantly reduced NLRP3 inflammasome activation, as indicated by reductions in the protein levels of NLRP3, ASC, and cleaved caspase-1 of approximately 42% (mean difference −1.86, 95% CI −2.69 to −1.03; P = 0.00068), 38% (mean difference −0.58, 95% CI −0.92 to −0.24; P = 0.0065), and 50% (mean difference −48.0, 95% CI −63.7 to −32.3; P = 2.9 × 10^-6^), respectively, compared with the M-HCC group. Similarly, BAI administration substantially reduced activation of the pyroptosis effector molecule, as indicated by a 25% reduction in full-length GSDMD (mean difference −0.61, 95% CI −1.07 to −0.15; P = 0.016) and a 59% reduction in GSDMD-N (mean difference −50.5, 95% CI −66.6 to −34.4; P = 1.3 × 10^-6^) compared with the M-HCC group (Figure 3D,E). BAI administration alone had no significant effect on any of these protein levels compared with the CTL group (P > 0.05; Table S3 in the Supplementary File). Consistent with the protein data, quantitative PCR analysis revealed that the mRNA expression of Nlrp3, Pycard (ASC), Caspase-1, and Gsdmd was significantly upregulated in the livers of M-HCC rats (Nlrp3: 95% CI for M-HCC 1.58 - 2.49; Pycard: 95% CI for M-HCC 2.19 - 2.95; Caspase-1: 4.42 ± 0.64 vs. 1.03 ± 0.21; Gsdmd: 2.91 ± 0.53 vs. 1.06 ± 0.17; all P < 0.01; Figure 3F – I). BAI treatment effectively downregulated the transcription of these genes, with expression levels in the M-HCC+BAI group being significantly lower than those in the M-HCC group (Nlrp3: 95% CI 1.18 - 1.79, P = 0.032 vs. M-HCC; Pycard: 95% CI 0.81 - 1.53, P < 0.001 vs. M-HCC; Caspase-1: 95% CI 2.03 - 2.82; Gsdmd: 95% CI 1.15 - 1.91; P < 0.05), and returning to levels comparable to those of the CTL group (P > 0.05 vs. CTL; Table S4 in the Supplementary File).

**Figure 3.**
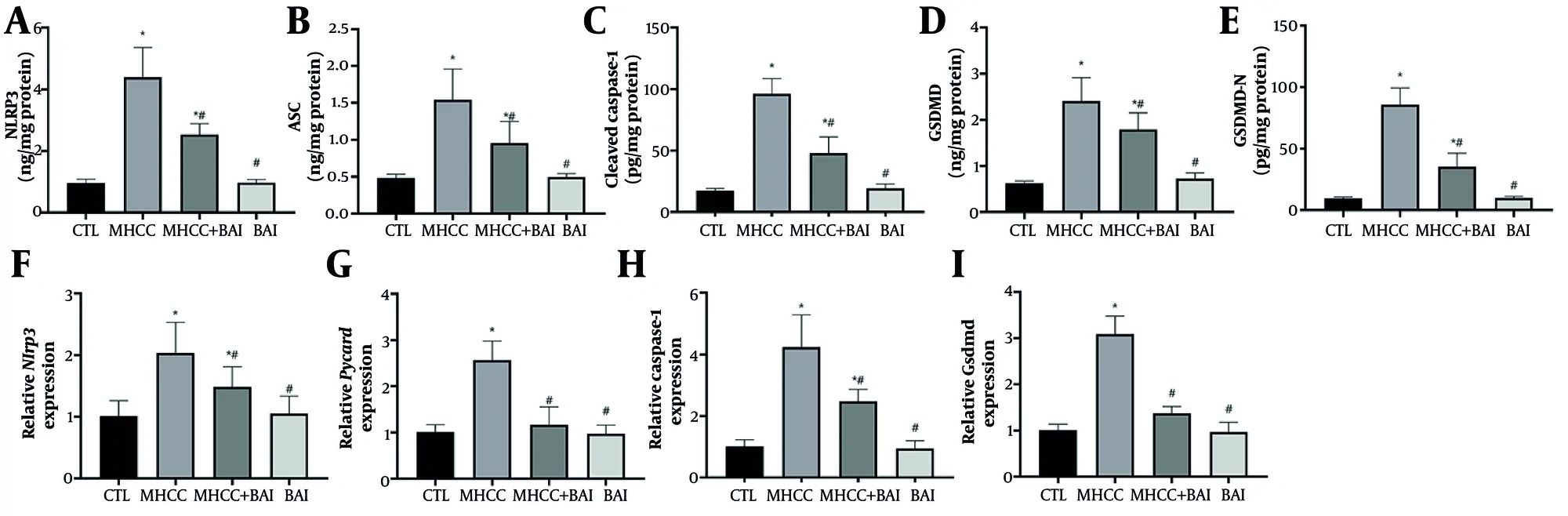
BAI inhibits the activation of the NLRP3 inflammasome and the pyroptosis pathway in vivo. (A–E) Hepatic protein levels of NLRP3, ASC, cleaved caspase-1, GSDMD, and the GSDMD-N fragment, as quantified by ELISA. (F–I) Hepatic mRNA expression levels of Nlrp3, Pycard (ASC), Caspase-1, and Gsdmd, normalized to Gapdh, as determined by RT-qPCR. Data are presented as mean ± SD (n = 8 per group). Statistical significance was determined by one-way ANOVA followed by Tukey's post hoc test. *: Significant difference vs. CTL; #: Significant difference vs. M-HCC; *#: Significant difference vs. both CTL and M-HCC groups. P < 0.05 was considered significant.

### 4.3. BAI Reduces Hepatic Inflammation and Fibrosis

Inhibition of the NLRP3 inflammasome and pyroptosis by BAI was accompanied by a significant decrease in the production of downstream inflammatory cytokines and hepatic fibrogenesis. In the M-HCC group, hepatic levels of the pyroptosis-related cytokines IL-1β and IL-18 were markedly elevated compared with the CTL group (IL-1β: mean difference 25.75 pg/mg protein, 95% CI 20.02 to 31.48; IL-18: mean difference 178.5 pg/mg protein, 95% CI 156.6 to 200.3), corresponding to approximately 3.0-fold increases for both cytokines (P < 0.001 for IL-1β and IL-18; Figure 4A,B). BAI treatment substantially reduced these elevations. IL-1β levels in the M-HCC+BAI group were significantly lower than those in the M-HCC group (mean difference 19.64 pg/mg protein, 95% CI 13.91 to 25.36; P < 0.0001). IL-18 levels were significantly lower than those in the M-HCC group (mean difference 117.2 pg/mg protein, 95% CI 95.32 to 139.0; P < 0.0001). These data represent reductions in IL-1β and IL-18 levels of 50.7% and 43%, respectively, compared with the untreated M-HCC group (both P < 0.01 vs. M-HCC), with values approaching those observed in CTL rats.

**Figure 4.**
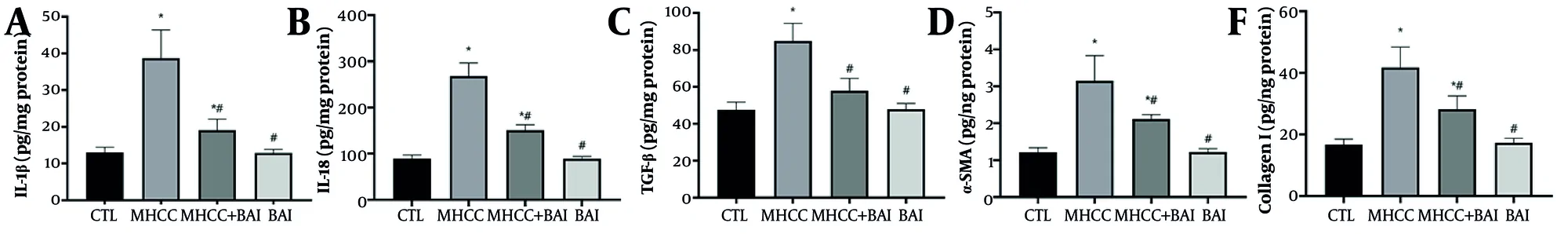
BAI reduces hepatic inflammation and fibrogenesis. (A, B) Hepatic protein levels of the pro-inflammatory cytokines IL-1β and IL-18. (C–E) Hepatic protein levels of the pro-fibrogenic cytokine TGF-β, the activated stellate cell marker α-smooth muscle actin (α-SMA), and collagen type I. All proteins were quantified by ELISA. Data are presented as mean ± SD (n = 8 per group). Statistical significance was determined by one-way ANOVA followed by Tukey's post hoc test. *: Significant difference vs. CTL; #: Significant difference vs. M-HCC; *#: Significant difference vs. both CTL and M-HCC groups. P < 0.05 was considered significant.

Concurrently, the BAI intervention significantly reduced activation of the pro-fibrogenic signaling cascade. Hepatic levels of the central fibrogenic cytokine TGF-β were markedly elevated in the M-HCC group compared with the CTL group (95% CI 28.54 to 46.06; P < 0.001; Figure 4C), corresponding to approximately a 1.78-fold increase (P < 0.001). BAI treatment significantly attenuated this increase, reducing TGF-β levels significantly (95% CI 18.27 to 35.78; P < 0.0001) in the M-HCC+BAI group, representing a marked decrease relative to the untreated M-HCC group and bringing values close to those of CTL rats. The reduced level of this fibrogenic cytokine was accompanied by decreased activation of hepatic stellate cells and reduced extracellular matrix deposition. Accordingly, hepatic α-smooth muscle actin (α-SMA) protein was markedly increased in the M-HCC group compared with the CTL group (95% CI for M-HCC 1.45 - 2.42; Figure 4D), corresponding to an approximate 2.6-fold elevation (P < 0.001). In the M-HCC+BAI group, α-SMA levels were significantly reduced (mean difference 1.04 ng/mg protein, 95% CI 0.56 to 1.52; P < 0.0001) compared with the M-HCC group. Similarly, hepatic collagen I content was significantly higher in M-HCC rats than in CTL rats (95% CI 19.43 to 30.64; Figure 4E), corresponding to an approximately 2.5-fold increase (P < 0.001). BAI treatment significantly reduced collagen I levels (mean difference 13.54 ng/mg protein, 95% CI 7.93 to 19.14; P < 0.0001) in the M-HCC+BAI group, representing a significant decrease relative to the M-HCC group. BAI alone did not significantly affect the levels of TGF-β, α-SMA, or collagen I compared with the CTL group (P > 0.05; Table S5 in the Supplementary File).

### 4.4. BAI Suppresses Indicators of Steatosis and Cellular Proliferation

Induction of the M-HCC model was associated with significant dysregulation of lipogenic pathways. Hepatic protein levels of SREBP-1c were elevated approximately 2.38-fold in the M-HCC group compared with CTL (95% CI 2.29 to 3.55; P < 0.0001; Figure 5A). Similarly, protein expression of PPARγ, a nuclear receptor critical for adipogenesis and lipid storage, was increased 3.57-fold in diseased livers (95% CI 1.98 to 2.59; P < 0.0001; Figure 5B). Treatment with BAI significantly mitigated these elevations, with SREBP-1c and PPARγ levels in the M-HCC+BAI group being significantly lower than those in the M-HCC group (For SREBP-1c: mean difference 1.85 ng/mg protein, 95% CI 1.22 to 2.49; P < 0.0001, For PPARγ: mean difference 1.19 ng/mg protein, 95% CI 0.88 to 1.49; P < 0.0001).

**Figure 5.**
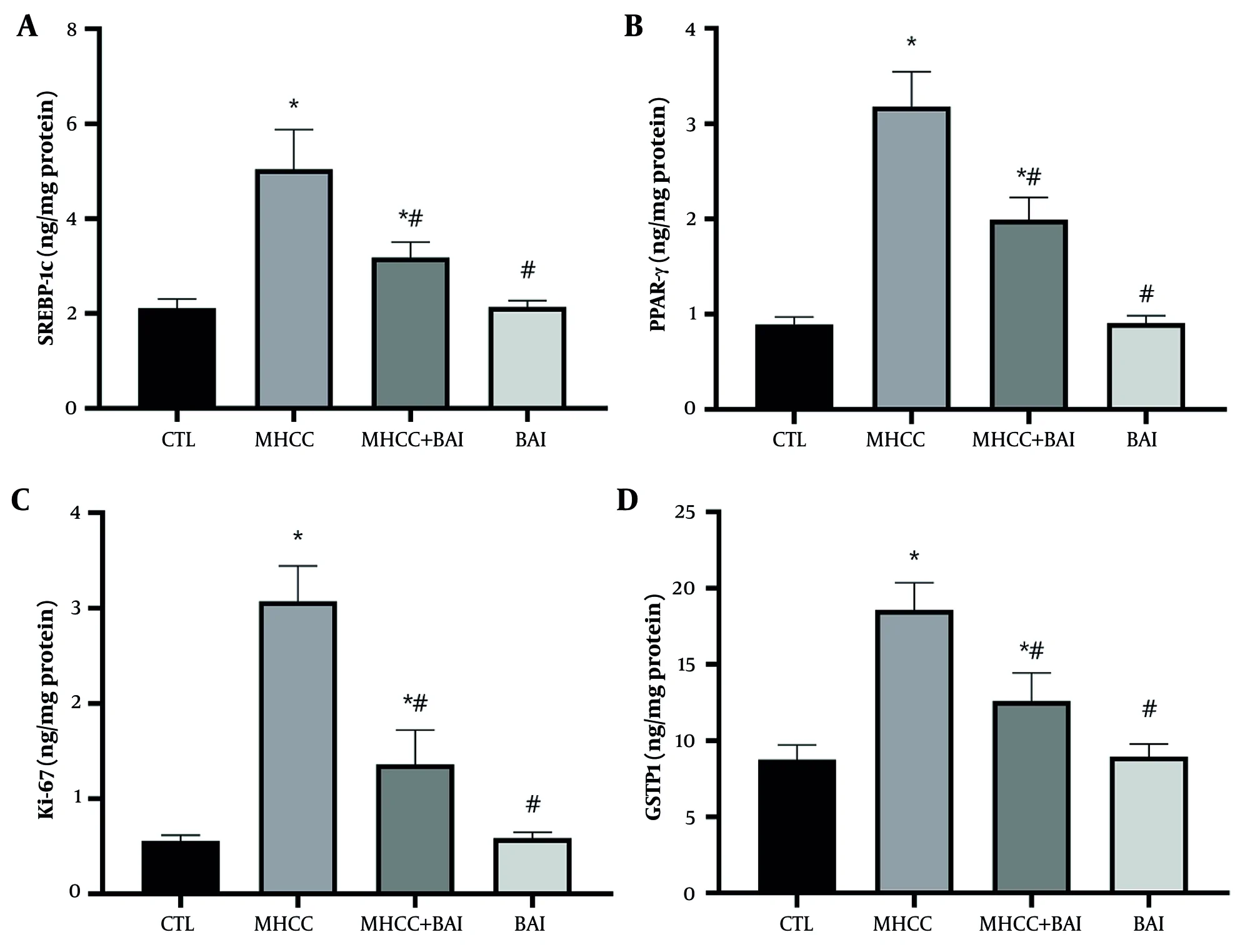
BAI suppresses markers of steatosis and cellular proliferation. (A, B) Hepatic protein levels of the lipogenic transcription factors SREBP-1c and PPARγ. (C) Hepatic protein level of the cellular proliferation marker Ki-67. (D) Hepatic protein level of glutathione S-transferase pi 1 (GSTP1). All proteins were quantified by ELISA. Data are presented as mean ± SD (n = 8 per group). Statistical significance was determined by one-way ANOVA followed by Tukey's post hoc test. *: Significant difference vs. CTL; #: Significant difference vs. M-HCC; *#: Significant difference vs. both CTL and M-HCC groups. P < 0.05 was considered significant.

As histopathological tumor quantification was not performed, these markers served as surrogate indicators of proliferative and preneoplastic changes. The findings showed that BAI exerted a marked antiproliferative effect within the preneoplastic hepatic milieu. The protein level of the nuclear proliferation marker Ki-67 was significantly elevated in the M-HCC group, showing a 5.48-fold increase relative to the CTL group (95% CI 2.16 to 2.87; P < 0.0001; Figure 5C). This hyperproliferative state was profoundly attenuated by BAI administration, which significantly reduced Ki-67 protein abundance (mean difference 1.71 ng/mg protein, 95% CI 1.35 to 2.07; P < 0.0001). Furthermore, we analyzed glutathione S-transferase pi 1 (GSTP1), a phase II detoxification enzyme frequently overexpressed during early hepatocarcinogenesis. Hepatic GSTP1 protein content was 2.12-fold higher in the M-HCC group than in CTL (95% CI 7.87 to 11.75; P < 0.0001; Figure 5D). BAI treatment significantly lowered GSTP1 levels, resulting in a marked reduction (mean difference 5.96 ng/mg protein, 95% CI 4.02 to 7.90; P < 0.0001) relative to the M-HCC group. Administration of BAI alone had no significant effect on the protein levels of SREBP-1c, PPARγ, Ki-67, or GSTP1 compared with the CTL group (Table S6 in the Supplementary File).

### 4.5. BAI Dose-Dependently Inhibits NLRP3 Inflammasome Activation and Pyroptosis in Lipotoxic HepG2 Cells

To validate the in vivo findings and determine whether BAI directly protects hepatocytes from NLRP3-mediated lipotoxic injury, HepG2 cells were exposed to a palmitate/oleate lipotoxic cocktail for 24 h. Lipotoxic stress significantly reduced cell viability (mean difference 35.29%, 95% CI 30.25 to 40.33; P < 0.0001) compared with untreated cells. Co-treatment with BAI dose-dependently improved viability to 71.0 ± 3.3% (95% CI: 67.6 - 74.4), 79.0 ± 3.6% (95% CI: 75.3 - 82.8), and 87.5 ± 3.8% (95% CI: 83.5 - 91.4) at 20, 40, and 80 µM, respectively (P < 0.0001 vs. LT). MCC950 significantly increased viability (95% CI: 80.7 - 85.7; Figure 6A), and its combination with BAI (80 µM) did not provide additional protection (95% CI: 84.0 - 88.4; P > 0.05). Importantly, BAI alone did not affect basal cell viability (95% CI: 95.6 - 101.9; P > 0.05 vs. control; Table S7 in the Supplementary File).

**Figure 6.**
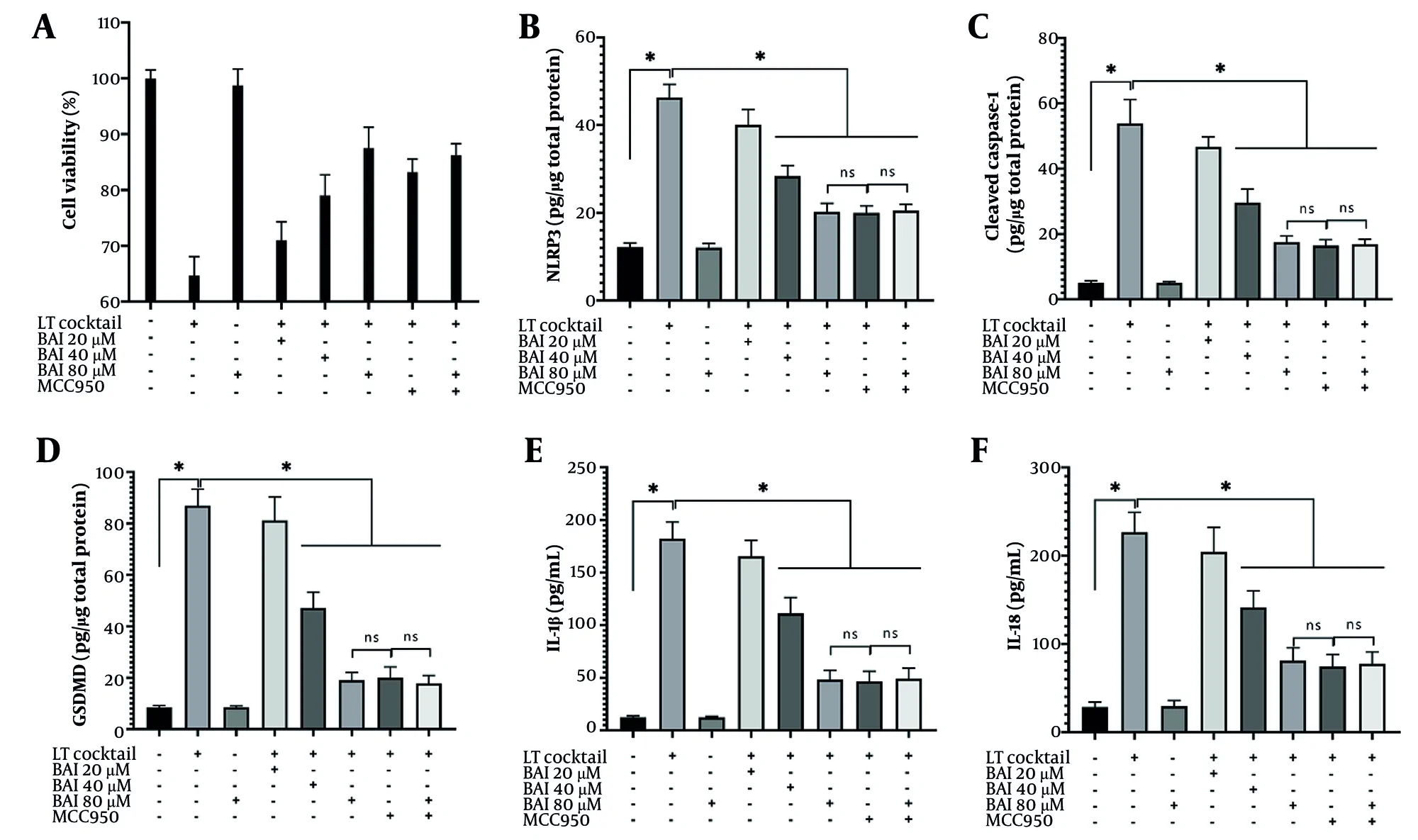
BAI dose-dependently inhibits NLRP3 inflammasome activation and pyroptosis in lipotoxic HepG2 cells. (A) Cell viability assessed by MTT assay after 24-h exposure to a lipotoxic cocktail (LT; 0.5 mM palmitate/1.0 mM oleate) with or without baicalein (BAI; 20, 40, 80 µM) and/or the NLRP3 inhibitor MCC950 (10 µM). (B–D) Cellular protein levels of NLRP3, cleaved caspase-1, and the GSDMD-N fragment as determined by ELISA. (E, F) Secreted levels of mature IL-1β and IL-18 in the culture supernatant measured by ELISA. All values are expressed as mean ± SD (n = 6 independent experiments, each performed in triplicate). Statistical significance was determined by one-way ANOVA followed by Tukey's post hoc test. * P < 0.05, ns: P > 0.05.

Lipotoxic stress robustly activated the NLRP3 inflammasome, as reflected by a nearly four-fold increase in NLRP3 protein levels (95% CI: 43.2 - 49.4 vs. 11.2 - 13.2; P < 0.0001; Figure 6B). BAI co-treatment attenuated this response in a dose-dependent manner, reducing NLRP3 expression to 40.1 ± 3.3 (95% CI: 36.6 - 43.6), 28.4 ± 2.4 (95% CI: 25.9 - 30.9), and 20.2 ± 2.0 (95% CI: 18.1 - 22.4) at 20 - 80 µM, corresponding to 13.3%, 38.7%, and 56.3% reductions vs. LT, respectively (P < 0.0001). Consistently, lipotoxicity markedly increased cleaved caspase-1 (95% CI: 46.3 - 61.3 vs. 4.6 - 5.7; P < 0.0001; Figure 6C). BAI suppressed caspase-1 activation in a concentration-dependent manner, decreasing levels to 46.7 ± 3.2, 29.6 ± 4.1, and 17.7 ± 2.0 (95% CI: 43.3 - 50.1, 25.3 - 33.9, and 15.6 - 19.8, respectively; P < 0.0001 for LT+BAI 80 vs. LT). MCC950 alone produced a comparable reduction (95% CI: 14.7 - 18.2), and its combination with high-dose BAI did not result in further inhibition (95% CI: 15.4 - 18.4; P > 0.05; Table S8 in the Supplementary File).

Lipotoxic stimulation markedly enhanced pyroptotic activation and inflammatory cytokine release in HepG2 cells. Compared with the control group, LT significantly increased GSDMD-N expression from 8.50 ± 0.71 (95% CI: 7.75 - 9.25) to 86.95 ± 6.40 (95% CI: 80.23 - 93.67), representing a 10.23-fold increase (P < 0.0001; Figure 6D). Similarly, IL-1β increased from 12.57 ± 1.05 (95% CI: 11.40 - 13.73) to 182.32 ± 15.73 (95% CI: 165.81 - 198.83), and IL-18 rose from 28.70 ± 5.49 (95% CI: 22.94 - 34.46) to 226.80 ± 21.08 (95% CI: 204.68 - 248.92), corresponding to 14.51-fold and 7.90-fold increases, respectively (P < 0.001; Figure 6E-F). BAI treatment attenuated these LT-induced changes in a dose-dependent manner. At 80 µM, BAI reduced GSDMD-N compared with LT alone (P < 0.0001) to 19.07 ± 2.95 (95% CI: 15.97 - 22.16), reduced IL-1β compared with LT alone (P < 0.0001) to 48.35 ± 8.39 (95% CI: 39.54 - 57.16), and reduced IL-18 compared with LT alone (P < 0.0001) to 81.02 ± 14.16 (95% CI: 66.16 - 95.88), corresponding to 78.07%, 73.48%, and 64.28% inhibition versus LT, respectively. MCC950 produced a comparable suppressive effect, and the combination of BAI80 with MCC950 did not confer an additional significant reduction, suggesting that baicalein mitigates lipotoxic pyroptosis primarily through suppression of inflammasome-dependent signaling (Table S9 in the Supplementary File).

## 5. Discussion

The attenuation of liver injury, reflected by marked reductions in serum ALT, AST, GGT, and ALP activities in the BAI-treated group, is consistent with the well-documented hepatoprotective profile of bioactive flavonoids (18). This effect may extend beyond membrane stabilization and may interfere with the cycle of hepatocyte injury and inflammatory recruitment during MASH progression. The concomitant reduction in serum HA levels further supports a beneficial effect on the fibrogenic microenvironment (19). Moreover, oxidative stress was attenuated in the BAI-treated group, as reflected by reduced lipid peroxidation and restored SOD activity. Given that oxidative stress is a major upstream activator of NLRP3 inflammasome assembly (20), the antioxidant potential of BAI may have contributed to its anti-inflammatory effects. This notion is supported by studies of other flavonoids, including naringenin and quercetin, in similar models (16, 21). The marked reduction in hepatic 8-OHdG levels further suggests alleviation of oxidative DNA damage (22, 23), which is particularly noteworthy given that persistent DNA damage links chronic inflammation to neoplasia. The protective effects of BAI likely reflect a multifaceted action, potentially involving direct ROS scavenging, cytochrome P450 modulation, or upregulation of endogenous antioxidant mechanisms.

Suppression of NLRP3 inflammasome and pyroptosis markers is a major finding of this study. Our results show that BAI treatment is associated with downregulation of the expression and activation of inflammasome complex molecules. In parallel, BAI treatment is associated with reduced GSDMD cleavage to its activated pore-forming fragment, GSDMD-N. This coordinated suppression suggests that BAI may interfere with components of this signaling cascade, potentially at a point upstream of caspase-1 activation. However, the precise molecular target(s) remain unidentified and require further biochemical investigation. The suppression of mature IL-1β and IL-18 functionally corroborates this inhibition, as these cytokines are direct products of caspase-1 activation (24, 25). The exact site of action remains speculative; possibilities include reduction of mitochondrial ROS (26), inhibition of TXNIP dissociation from thioredoxin (27), or direct interference with NLRP3/ASC oligomerization. The MCC950 experiment showed that BAI's effects involve the NLRP3 pathway, as the inhibitor did not produce an additive effect. However, this finding supports pathway involvement without establishing direct molecular targeting of NLRP3. Furthermore, BAI may modulate other pathways independent of NLRP3, such as the NF-κB pathway, which is also implicated in inflammasome priming and subunit expression (28).

The downstream consequences of NLRP3 inflammasome and pyroptosis inhibition were reflected in the significant attenuation of hepatic inflammation and fibrosis. The reductions in mature IL-1β and IL-18 levels indicate suppression of the highly inflammatory environment that perpetuates hepatocellular injury and non-parenchymal cell activation (29). BAI also reduced the levels of the pivotal fibrogenic cytokine TGF-β, the hepatic stellate cell activation marker α-SMA, and the matrix deposition marker collagen I. These findings suggest that BAI disrupts the crucial link between inflammation and fibrogenesis. This finding is consistent with hepatic stellate cell activation driven by pyroptosis-induced inflammation. Although these findings accord with previous studies implicating other NLRP3 inhibitors, such as nicotinic acid in this model, in fibrosis attenuation, they cannot establish whether BAI attenuates fibrosis through direct or indirect mechanisms. BAI also suppressed lipogenic transcription factors such as SREBP-1c and the adipogenic nuclear receptor PPARγ. These findings suggest that BAI favorably affects dysregulated hepatic lipid metabolism, a key component of the MASH disease cascade (30, 31). This anti-steatotic activity may contribute to the overall improvement of the hepatic microenvironment, potentially reducing lipotoxicity-induced inflammasome activity (30, 31). Importantly, the significant reductions in the proliferation marker Ki-67 and the pre-neoplastic marker GSTP1 have translational value. These findings suggest that BAI may modulate the inflammatory and metabolic components of the disease cascade and is also associated with reduced dysregulated compensatory proliferation, a key feature of carcinogenesis (32). Overall, the data suggest that BAI exerts multifaceted activity across the interconnected disease axes of steatosis, inflammation, fibrosis, and proliferation, which may offer an advantage over single-target therapies in the multifactorial disease cascade of MASH-driven hepatocarcinogenesis.

### 5.1. Study Limitations

Several limitations should be acknowledged. First, although the M-HCC model used here has been previously characterized to produce HCC at 16 - 18 weeks, we did not perform direct histopathological tumor quantification; our endpoints are therefore limited to molecular surrogate markers (Ki-67 and GSTP1), and the term "hepatocarcinogenesis" refers to early molecular events rather than confirmed HCC. Second, while rodent models are widely used for studying MASH-driven liver pathology, they do not fully recapitulate human disease complexity, and the HepG2 cell line, used for mechanistic validation, is a cancer cell line rather than normal hepatocytes; thus, our findings require validation in more physiologically relevant models. Third, the observed reductions in NLRP3 pathway components and pyroptosis markers are associative rather than causal; the precise molecular target of BAI remains unidentified, and future studies employing genetic loss-of-function approaches and Western blot validation would strengthen mechanistic interpretation. Fourth, we did not monitor metabolic parameters such as body weight, glucose, or insulin, which could provide additional insight into the degree of metabolic dysfunction. Finally, BAI was administered prophylactically; its therapeutic efficacy in established disease remains unknown. Despite these limitations, the consistent pattern of effects across in vivo and in vitro models provides a robust foundation for further investigation.

### 5.2. Conclusions

Collectively, the findings from this integrated in vivo and in vitro study indicate that baicalein treatment can attenuate multiple pathological hallmarks associated with MASH-driven hepatocarcinogenesis, including hepatic injury, oxidative stress, steatosis, inflammation, and fibrosis. The protective effects were consistently associated with inhibition of the NLRP3 inflammasome and the subsequent pyroptosis pathway, leading to reduced levels of active inflammatory cytokines and proliferation markers. These results provide mechanistic insight into baicalein's potential as a pleiotropic agent affecting multiple biological pathways in this context. While these findings position BAI as a promising candidate for further investigation into its potential to modulate early hepatocarcinogenic events, they do not constitute direct evidence of HCC prevention. Studies incorporating histological tumor burden, incidence, and histopathological confirmation are required to determine whether baicalein exerts genuine chemopreventive or anti-HCC effects. Further research is also needed to elucidate its precise molecular targets, evaluate long-term efficacy and safety, and validate these findings in more complex model systems and, ultimately, clinical settings.

ijpr-25-1-171126-s001.pdf

## Data Availability

The dataset presented in the study is available on request from the corresponding author during submission or after publication.
