# Supplementary material for: Baicalein Attenuates Early Molecular and Proliferative Changes in MASH-Driven Hepatocarcinogenesis by Inhibiting the NLRP3 Inflammasome and Pyroptosis Pathway
Source: Iran J Pharm Res. 2026 Sep 9;25(1):e171126. doi: 10.5812/ijpr-171126 (PMC13647231; doi:10.5812/ijpr-171126)
Supplement: Supplementary file 1 [file ijpr-25-1-171126-s001.pdf]

Biomarker	Group	Mean ± SD	95% CI
**GGT**	CTL	4.06 ± 0.72	3.46-4.66
	MHCC	24.03 ± 5.12	19.74-28.31
	MHCC+BAI	8.24 ± 1.12	7.30-9.17
	BAI	5.91 ± 1.18	4.92-6.90
**AST**	CTL	88.89 ± 3.43	86.02-91.76
	MHCC	307.05 ± 37.26	275.90-338.20
	MHCC+BAI	175.21 ± 27.09	152.56-197.86
	BAI	102.00 ± 13.14	91.02-112.98
**ALP**	CTL	133.00 ± 4.82	128.97-137.03
	MHCC	230.08 ± 17.42	215.51-244.64
	MHCC+BAI	156.95 ± 14.80	144.58-169.32
	BAI	138.85 ± 9.66	130.78-146.92
**ALT**	CTL	41.26 ± 2.38	39.27-43.25
	MHCC	191.36 ± 14.96	178.85-203.87
	MHCC+BAI	104.95 ± 10.26	96.37-113.53
	BAI	42.73 ± 5.60	38.04-47.32

Supplementary Table S1. Serum biochemical indices in rats from the control, model, and baicalein-treated groups. Values are presented as mean ± SD (95% CI) for n = 8 rats per group. Rats were assigned to CTL, BAI, M-HCC, and M-HCC+BAI groups. Serum ALT, AST, ALP, and GGT were measured using standardized colorimetric methods. M-HCC was induced using a hepatopathogenic diet, carbon tetrachloride (CCl_4), and diethylnitrosamine (DEN). BAI indicates baicalein, administered orally at 50 mg/kg/day. Statistical comparisons were performed in blinded analyses.

Biomarker	Group	Mean ± SD	95% CI
**8-OHDG**	CTL	2.01 ± 0.13	1.90-2.12
	MHCC	4.14 ± 0.56	3.50-4.78
	MHCC+BAI	2.84 ± 0.37	2.53-3.15
	BAI	2.04 ± 0.13	1.93-2.15
**HA**	CTL	45.11 ± 3.05	42.55-47.66
	MHCC	119.14 ± 9.25	111.39-126.89
	MHCC+BAI	65.39 ± 8.24	58.49-72.30
	BAI	53.21 ± 10.06	44.78-61.65
**MDA**	CTL	1.96 ± 0.12	1.86-2.06
	MHCC	4.99 ± 0.39	4.66-5.32
	MHCC+BAI	3.03 ± 0.75	2.41-3.66
	BAI	1.97 ± 0.08	1.90-2.05
**SOD**	CTL	33.80 ± 1.65	32.42-35.19
	MHCC	18.16 ± 1.23	17.13-19.19
	MHCC+BAI	27.98 ± 3.94	24.68-31.28
	BAI	34.00 ± 1.46	32.78-35.22

Supplementary Table S2. Liver injury and oxidative stress–related markers in rats from the control, model, and baicalein-treated groups. Values are presented as mean ± SD (95% CI) for n = 8 rats per group. Biomarkers include 8-OHdG, HA, MDA, and SOD, measured in serum or liver tissue as described in the Methods. 8-OHdG was quantified by competitive ELISA and expressed as pg/mg total protein. HA was measured by ELISA, while MDA and SOD were assessed in liver tissue using standard biochemical assays. Rats were allocated to CTL, BAI, M-HCC, and M-HCC+BAI groups; BAI was administered orally at 50 mg/kg/day.

Biomarker	Group	Mean ± SD	95% CI
**ASC**	CTL	0.48 ± 0.05	0.44–0.53
	MHCC	1.54 ± 0.42	1.20–1.89
	MHCC+BAI	0.96 ± 0.29	0.71–1.20
	BAI	0.50 ± 0.05	0.45–0.54
**GSDMD**	CTL	0.62 ± 0.05	0.58–0.67
	MHCC	2.40 ± 0.51	1.98–2.83
	MHCC+BAI	1.79 ± 0.36	1.49–2.09
	BAI	0.73 ± 0.12	0.63–0.83
**GSDMD-N**	CTL	9.66 ± 0.99	8.83–10.49
	MHCC	85.88 ± 13.40	74.67–97.08
	MHCC+BAI	35.39 ± 11.07	26.14–44.64
	BAI	10.12 ± 1.04	9.26–10.99
**NLRP3**	CTL	0.96 ± 0.12	0.86–1.06
	MHCC	4.39 ± 0.97	3.58–5.20
	MHCC+BAI	2.53 ± 0.35	2.24–2.83
	BAI	0.97 ± 0.10	0.89–1.05
**Cleaved Caspase-1**	CTL	17.38 ± 1.80	15.87–18.88
	MHCC	96.04 ± 12.40	85.67–106.41
	MHCC+BAI	47.96 ± 13.19	36.93–58.99
	BAI	19.40 ± 3.43	16.53–22.27

Supplementary Table S3. Pyroptosis-related markers in liver tissue from the control, model, and baicalein-treated groups. Values are presented **as** mean ± SD (95% CI) for n = 8 rats per group. Liver tissue levels of ASC, GSDMD, GSDMD-N, NLRP3, and cleaved caspase-1 were measured by ELISA and normalized to total protein content. Rats were distributed into CTL, BAI, M-HCC, and M-HCC+BAI groups. M-HCC was established using a hepatopathogenic diet, CCl_4, and DEN, and BAI was given orally at 50 mg/kg/day.

Biomarker	Group	Mean ± SD	95% CI
Pycard	CTL	1.01 ± 0.16	0.88–1.15
	MHCC	2.54 ± 0.38	2.22–2.85
	MHCC+BAI	1.14 ± 0.33	0.87–1.42
	BAI	0.98 ± 0.17	0.84–1.12
Nlrp3	CTL	1.00 ± 0.26	0.78–1.21
	MHCC	2.02 ± 0.54	1.57–2.47
	MHCC+BAI	1.42 ± 0.42	1.07–1.77
	BAI	1.00 ± 0.29	0.76–1.24
Caspase-1	CTL	1.03 ± 0.26	0.81–1.24
	MHCC	4.33 ± 1.19	3.33–5.32
	MHCC+BAI	2.58 ± 0.53	2.14–3.02
	BAI	0.99 ± 0.25	0.78–1.20
Gsdmd	CTL	1.04 ± 0.16	0.90–1.17
	MHCC	3.17 ± 0.34	2.88–3.45
	MHCC+BAI	1.39 ± 0.14	1.28–1.51
	BAI	0.97 ± 0.17	0.82–1.12

Supplementary Table S4. Relative mRNA expression levels of pyroptosis-related genes in liver tissue from the control, model, and baicalein-treated groups. Values are presented as mean ± SD (95% CI) for n = 8 rats per group. Hepatic mRNA expression of *Pycard* (ASC), *Nlrp3*, *Casp1*, and Gsdmd was quantified by quantitative real-time PCR (qPCR) and expressed as relative expression using the 2^-ΔΔCt method. Rats were assigned to CTL, BAI, M‑HCC, and M‑HCC+BAI groups. BAI indicates baicalein administered orally at 50 mg/kg/day.

Biomarker	Group	Mean ± SD	95% CI

**IL-18**	CTL	90.25 ± 7.23	84.21–96.29
	MHCC	267.05 ± 28.40	243.30–290.80
	MHCC+BAI	151.61 ± 10.68	142.68–160.54
	BAI	90.08 ± 4.43	86.37–93.78
**IL-1β**	CTL	13.04 ± 1.36	11.91–14.18
	MHCC	38.49 ± 7.31	32.38–44.60
	MHCC+BAI	19.10 ± 2.86	16.71–21.49
	BAI	12.91 ± 0.96	12.11–13.71
**TGF-β**	CTL	48.28 ± 4.01	44.93–51.63
	MHCC	87.08 ± 8.90	79.64–94.52
	MHCC+BAI	58.73 ± 6.22	53.53–63.93
	BAI	47.64 ± 3.00	45.13–50.15
**α-SMA**	CTL	1.21 ± 0.13	1.10–1.32
	MHCC	3.40 ± 0.72	2.80–4.00
	MHCC+BAI	2.11 ± 0.11	2.02–2.21
	BAI	1.22 ± 0.09	1.15–1.30
**Collagen I**	CTL	17.15 ± 1.45	15.94–18.36
	MHCC	41.60 ± 7.17	35.60–47.60
	MHCC+BAI	27.36 ± 4.10	23.93–30.78
	BAI	17.31 ± 1.32	16.21–18.42

Supplementary Table S5. Fibrosis-related markers in liver tissue from the control, model, and baicalein-treated groups. Values are presented as mean ± SD (95% CI) for n = 8 rats per group. Liver tissue levels of IL-18, IL-1β, TGF-β, α-SMA, and collagen I were determined by ELISA and normalized to total protein content. Rats were allocated to CTL, BAI, M-HCC, and M-HCC+BAI groups. M-HCC was induced using a hepatopathogenic diet, CCl_4, and DEN, and BAI was administered orally at 50 mg/kg/day.

Biomarker	Group	Mean ± SD	95% CI
**SREBP-1c**	CTL	2.12 ± 0.18	1.97–2.28
	MHCC	5.03 ± 0.82	4.34–5.71
	MHCC+BAI	3.18 ± 0.32	2.91–3.44
	BAI	2.14 ± 0.14	2.02–2.25
**PPARγ**	CTL	0.89 ± 0.08	0.82–0.95
	MHCC	3.18 ± 0.35	2.89–3.47
	MHCC+BAI	2.00 ± 0.25	1.79–2.20
	BAI	0.91 ± 0.08	0.84–0.97
**KI-67**	CTL	0.56 ± 0.06	0.51–0.61
	MHCC	3.07 ± 0.37	2.76–3.38
	MHCC+BAI	1.36 ± 0.33	1.09–1.64
	BAI	0.58 ± 0.06	0.53–0.63
**GSTP1**	CTL	8.76 ± 0.99	7.94–9.59
	MHCC	18.57 ± 1.87	17.01–20.13
	MHCC+BAI	12.61 ± 1.69	11.20–14.03
	BAI	8.95 ± 0.82	8.26–9.63

Supplementary Table S6. Lipid metabolism and proliferation markers in liver tissue from the control, model, and baicalein-treated groups. Values are presented as mean ± SD (95% CI) for n = 8 rats per group. Liver tissue levels of SREBP-1c, PPARγ, Ki-67, and GSTP1 were measured by ELISA and normalized to total protein content. Rats were assigned to CTL, BAI, M-HCC, and M-HCC+BAI groups. BAI indicates baicalein, administered orally at 50 mg/kg/day.

**Group**	**Mean ± SD**	**95% CI**
Control	99.9 ± 1.51	98.3-101.5
LT	64.63 ± 3.25	61.2-68.0
BAI 80 μM	98.73 ± 3.00	95.6-101.9
LT + BAI 20	71.00 ± 3.25	67.6-74.4
LT + BAI 40	79.03 ± 3.58	75.3-82.8
LT + BAI 80	87.45 ± 3.77	83.5-91.4
LT + MCC950	83.20 ± 2.42	80.7-85.7
LT + BAI 80 + MCC950	86.23 ± 2.10	84.0-88.4

Supplementary Table S7. Effects of BAI and the NLRP3 inhibitor MCC950 on HepG2 cell viability under lipotoxic conditions. Cell viability was determined by MTT assay after 24 h exposure to a palmitate/oleate lipotoxic cocktail in the presence or absence of BAI (20–80 μM) or MCC950. Values are presented as mean ± SD with corresponding 95% confidence intervals (CI) from six independent replicates (n = 6). Percent viability is expressed relative to untreated control cells.

Group	NLRP3		Cleaved Caspase-1	
	**Mean ± SD**	**95% CI**	**Mean ± SD**	**95% CI**
Control	12.17 ± 0.96	11.2-13.2	5.08 ± 0.57	4.5-5.7
LT	46.28 ± 3.04	43.2-49.4	53.83 ± 6.54	46.3-61.3
BAI 80	12.10 ± 0.93	11.2-13.2	5.12 ± 0.35	4.8-5.4
LT + BAI 20	40.12 ± 3.36	36.6-43.6	46.73 ± 3.33	43.3-50.1
LT + BAI 40	28.38 ± 2.37	25.9-30.9	29.62 ± 3.96	25.3-33.9
LT + BAI 80	20.23 ± 1.94	18.1-22.4	17.52 ± 2.05	15.6-19.8
LT + MCC950	20.02 ± 1.52	18.3-21.8	16.45 ± 1.82	14.7-18.2
LT + BAI 80 + MCC950	20.53 ± 1.41	19.0-22.1	16.87 ± 1.60	15.4-18.4

Supplementary Table S8. Effects of BAI and MCC950 on NLRP3 inflammasome activation in lipotoxic HepG2 cells. Quantitative densitometric analysis of ELISA for NLRP3 and cleaved caspase-1 following 24 h lipotoxic stimulation with or without BAI (20–80 µM) or MCC950. Values are reported as mean ± SD with 95% confidence intervals (CI) from six independent replicates (n = 6). Percent reduction represents the decrease relative to the lipotoxic (LT) group.

Group	GSDMD-N		IL-1β		IL-18	
	Mean ± SD	95% CI	Mean ± SD	95% CI	Mean ± SD	95% CI
Control	8.50 ± 0.71	7.75–9.25	12.57 ± 1.05	11.47-13.67	28.70 ± 5.49	22.94-34.46
LT	86.95 ± 6.40	80.23–93.67	182.32 ± 15.73	165.81-198.83	226.80 ± 21.08	204.68-248.92
BAI80	8.55 ± 0.53	7.99–9.11	12.47 ± 0.91	11.5-13.4	29.57 ± 6.33	22.9-36.2
LT+BAI20	81.22 ± 9.10	71.66–90.77	165.48 ± 14.72	149.5-181.5	204.42 ± 27.73	175.3-233.5
LT+BAI40	47.17 ± 6.06	40.81–53.53	111.27 ± 14.26	96.3-126.2	141.58 ± 17.78	122.9-160.2
LT+BAI80	19.07 ± 2.95	15.97–22.16	48.35 ± 8.39	39.54-57.16	81.02 ± 14.16	66.16-95.88
LT+MCC950	20.03 ± 4.17	15.65–24.41	46.72 ± 10.01	34.69-58.75	74.58 ± 11.85	60.77-88.39
LT+BAI80+MCC950	17.88 ± 2.94	14.80–20.96	49.30 ± 11.44	34.18-64.42	77.62 ± 12.34	62.62-92.62

Supplementary Table S9. Pyroptosis-related markers in HepG2 cells after lipotoxic stimulation and baicalin treatment. ELISA measurements of GSDMD-N, IL-1β, and IL-18 in HepG2 cells treated with lipotoxic stimulus (LT) and baicalin (BAI; 20–80 μM) with or without MCC950 (NLRP3 inhibitor). Data are presented as mean ± SD (n = 6) and 95% confidence intervals (CI).
